# Inverted stress- induced cardiomyopathy as a unusual variant of acute heart failure after cesarean delivery- a case report

**DOI:** 10.1186/s12872-016-0253-z

**Published:** 2016-04-29

**Authors:** Anna Ledakowicz-Polak, Jarosław Bartodziej, Agata Majos, Marzenna Zielińska

**Affiliations:** Intensive Cardiac Therapy Clinic, Department of Cardiology and Cardiosurgery, Medical University, Pomorska 251, 92-213 Lodz, Poland; Department of Anaesthesiology and Intensive Care, Jonscher Municipal Hospital, Lodz, Poland; Department of Radiology, Medical University, Lodz, Poland

**Keywords:** Inverted stress- induced cardiomyopathy, Acute heart failure, Cesarean delivery

## Abstract

**Background:**

Stress- induced cardiomyopathy is acute, reversible left ventricle mainly apical dysfunction in patients with normal coronary angiography. Rarely it regards basal segments, therefore defined as “inverted stress- induced cardiomyopathy”. While classic form mostly affects postmenopausal women, inverted variant occurs essentially in younger females, always triggered by stress. It can also develop after medical procedures and surgery. Herein we report such unique case of 36- year old woman after cesarean delivery.

**Case presentation:**

A 36- year- old white woman at 40 week of gestation was admitted to hospital for elective repeated cesarean delivery. During caesarean delivery under spinal anaesthesia a previously healthy woman became hypotensive, requiring ephedrine to maintain her blood pressure. Three hours after delivery the patient presented acute heart failure and pulmonary oedema. Due to low blood pressure she demanded the administration of inotropic agents. Owing to respiratory failure and gradual deterioration of consciousness, mechanical ventilation was applied. Results of additional tests finally excluded pulmonary thromboembolism and acute coronary syndrome. The transthoracic echocardiography revealed severe left ventricular systolic dysfunction, ejection fraction 30 % with hypokinesis of the mid and basal segments of posterior, anterior and lateral wall with preserved contractility of the apical segments. The diagnosis of inverted stress- induced cardiomyopathy was set upon the overall clinical data. Both echocardiography and magnetic resonance imaging performed on the fifth day showed complete recovery of myocardial function. The patient was discharged after 15 days in good overall condition. At 12- month follow- up she remained asymptomatic with no echocardiographic abnormalities.

**Conclusions:**

Inverted stress- induced cardiomyopathy may occur in postpartum period, especially in combination with spinal anesthesia and adrenergic stimulants administration. The clinical awareness and multimodality imaging of possible diagnosis and further management of this unexpected variant of acute heart failure after caesarean delivery is required.

## Background

Stress- induced cardiomyopathy (SIC) also known as “takotsubo cardiomyopathy” is characterized by reversible left ventricular dysfunction with chest symptoms (pain or dyspnea), electrocardiogram changes that mimic those of acute coronary syndrome (ACS) and minor elevation in serum levels of cardiac enzymes in patients with normal coronary angiography [1]. While echocardiography revealed apical ballooning in the majority of cases, a small part of patients present with basal dysfunction also defined as “inverted or reverse SIC”. Inversely to classic form of SIC, which mainly affects postmenopausal women, the reverse variant of SIC occurs essentially in younger females and is always triggered by emotional or physical stress [2].

We report an extremely rare case of inverted SIC which occurred in young women after cesarean delivery.

## Case presentation

**Fig. 1:**
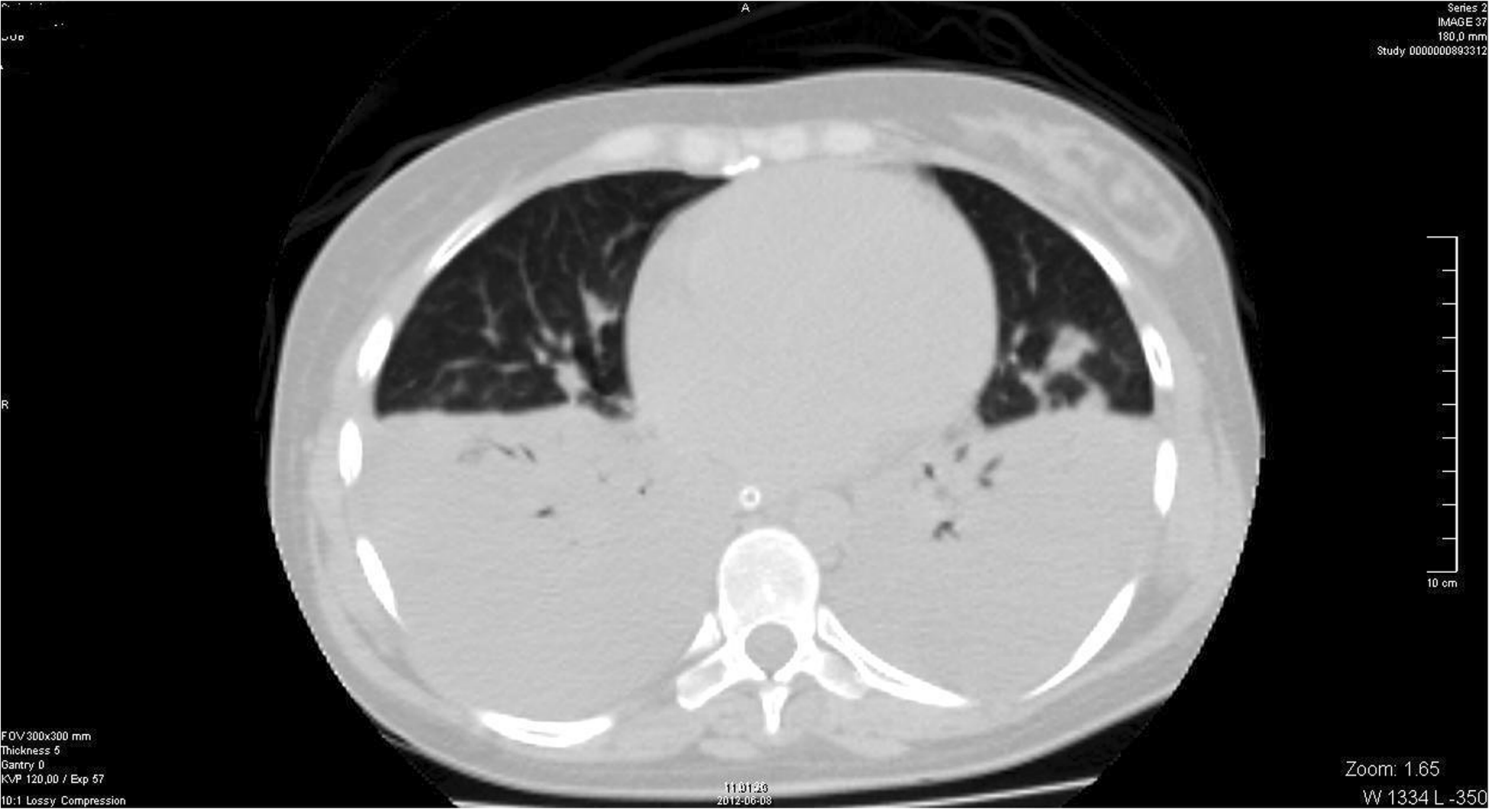
Chest computed tomography demonstrating severe pulmonary oedema

**Fig. 2:**
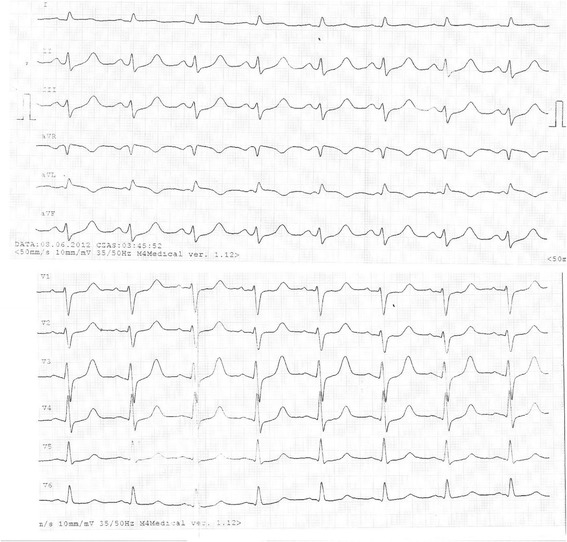
Electrocardiogram

**Fig. 3:**
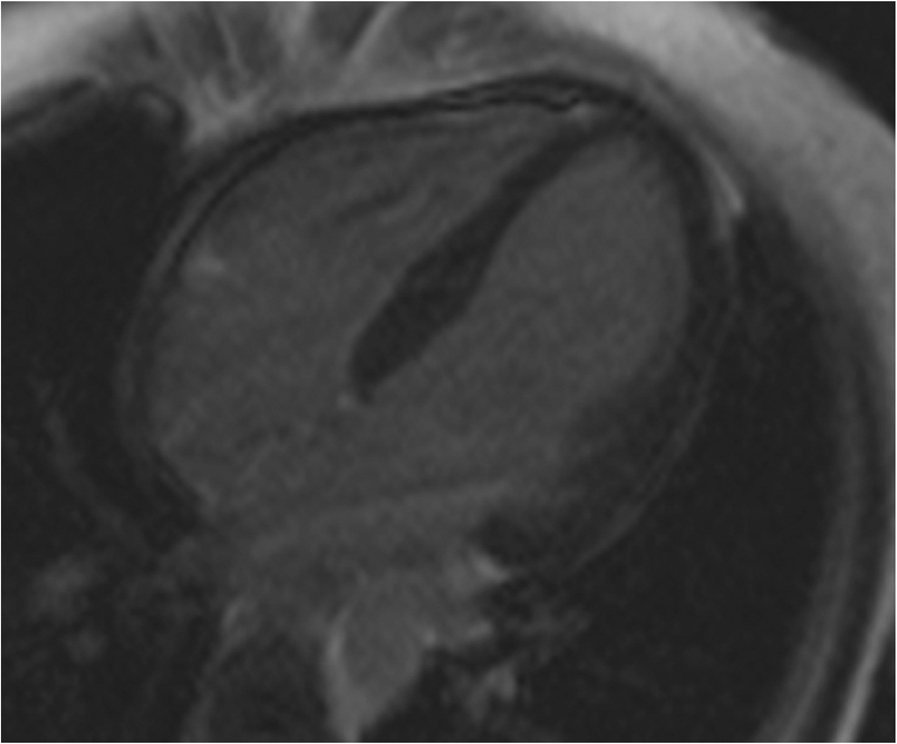
Cardiac magnetic resonance imaging with late gadolinium enhancement in four chamber view, without evidence of tissue abnormalities (scars, patchy fibrosis)

**Fig. 4:**
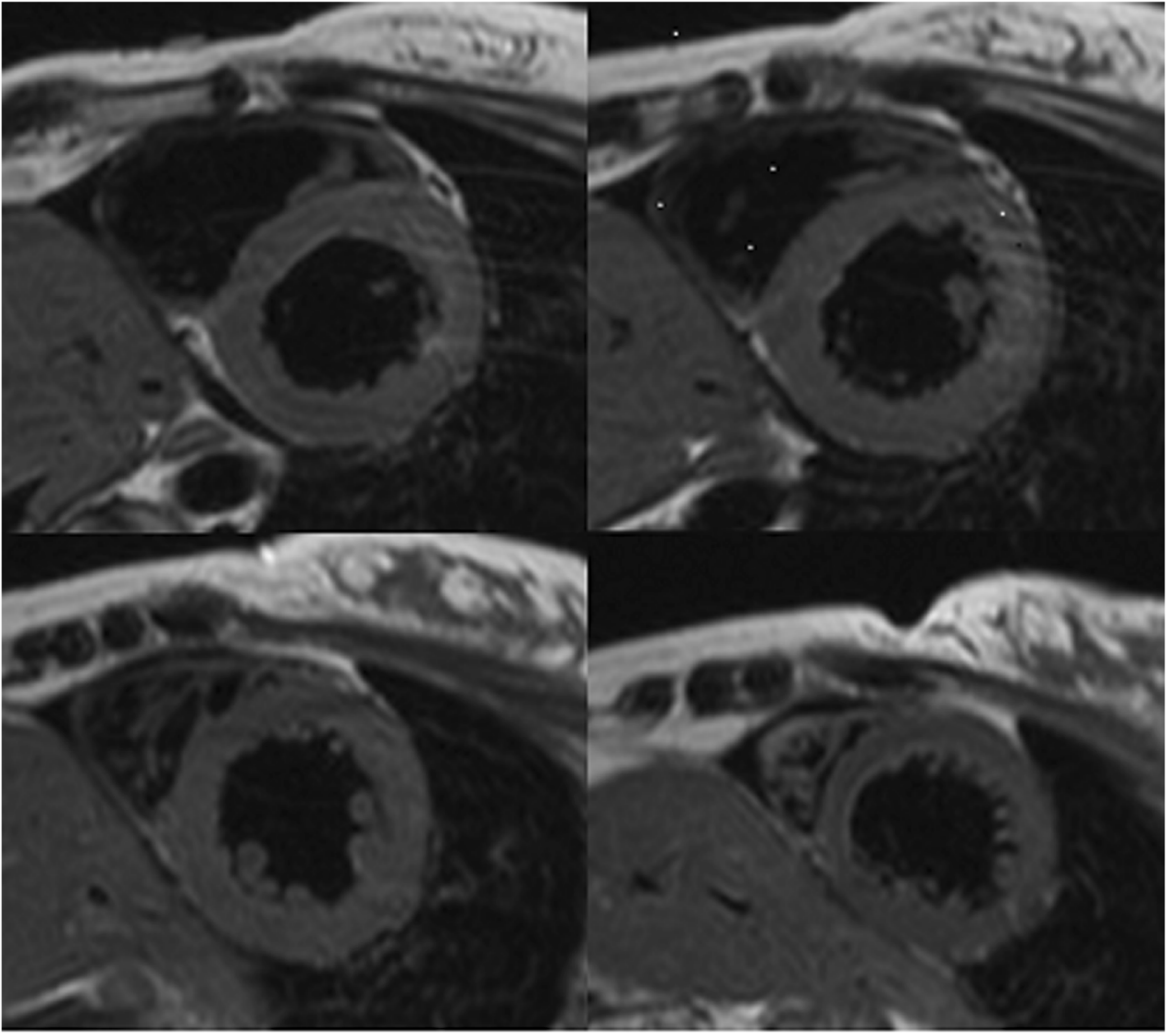
Short-axis, dark- blood T2- weighted cardiac magnetic resonance imaging demonstrating no signs of myocardial edema

A 36- year- old white woman at 40 week of gestation was admitted to a local obstetric clinic for elective repeated cesarean delivery due to slanting position of the fetus. Both her previous and current pregnancy were uncomplicated. Her past medical history was unremarkable and no family history of cardiac disease was reported. The caesarean delivery was performed following the administration of standard spinal anesthesia. During surgery the patient became hypotensive and ephedrine was injected to maintain her blood pressure in the normal range. Three hours after delivery of healthy male infant, the patient complained of nausea, increasing dyspnoea and palpitations. On physical examination tachycardia with ventricular extra systoles and pulmonary rales were detected. Her blood pressure was 80/40 mmHg and demanded the administration of inotropic agents (initially continuous infusion of norepinephrine 0.5 mg/h followed by dobutamine 7 μg/kg/min). Owing to worsening of oxygen saturation up to 70 % and gradual deterioration of consciousness, mechanical ventilation was applied and the patient was transferred to intensive care unit. Chest X ray indicated pulmonary congestion. Emergency computer tomography excluded pulmonary thromboembolism (PE) and confirmed severe pulmonary oedema (Fig. 1). The patient received loop diuretic, furosemide, at initial daily dose 80 mg, which was progressively reduced. The diuretic was discontinued after 12 days of treatment. The electrocardiogram disclosed sinus tachycardia with ST- segment elevation of 1,5 mm with negative T waves in aVL and ST- segment depression of 1 mm in II, III, aVF, V5-V6 (Fig. 2). Laboratory tests showed elevated troponin up to 908 pg/ml (normal value <14 pg/ml), NT- pro BNP 6236 pg/ml (normal value <125 pg/ml). The bedside transthoracic echocardiography (TTE) revealed severe left ventricular (LV) systolic dysfunction. Therefore the patient was transferred to Intensive Cardiac Therapy Clinic. Repeated TTE showed LV ejection fraction 30 % with hypokinesis of the mid and basal segments of posterior, anterior and lateral wall with preserved contractility of the apical segments. Urgent coronary angiography presented normal coronary arteries. The diagnosis of inverted stress- induced cardiomyopathy was set upon the overall clinical data. After 3 days the patient was weaned from respirator and extubated. Following hemodynamic improvement, inotropic agents were tapered gradually. Both TTE and magnetic resonance imaging (Figs. 3 and 4) performed on the fifth day showed complete recovery of myocardial function. The patient was discharged after 15 days in good overall condition. At 12- month follow- up she remained asymptomatic with no echocardiographic abnormalities.

## Discussion

It has been shown that SIC predominantly affects postmenopausal woman [1]. Although several cases of SIC in young woman have already been described, pregnancy associated SIC (P-SIC) appears to be rather unusual [3–7].

The pathophysiological mechanism of SIC is still not completely understood. Several possibilities have been discussed but increased levels of catecholamine and vasoconstrictive substances appear to play crucial role in triggering SIC [8]. It can be defined as a catecholamine- triggered myocyte injury caused by catecholamine- induced microvascular spasm or by direct catecholamine- associated myocardial toxicity. Cardiac troponins are elevated in almost all patients with SIC. However the elevation in serum troponin is disproportional in comparison to the extension of the akinetic myocardial regions. The pathophysiology of wall motion abnormalities in SIC is not well established. Reversible ventricular dysfunction may be also the result of epicardial coronary artery spasm and consequently regionally stunned myocardium [9]. This extended stunning is probably correlated with only slight elevation of cardiac necrosis markers. Moreover it has been reported that the risk of SIC can be reduced by blocking α- and β- adrenergic receptors [10, 11]. Furthermore, a difference in the distribution and density of adrenergic receptors in myocardium are responsible for the variance in SIC appearance in either the apical or mid- ventricular wall. Inverted SIC occurs mainly in younger females with higher prevalence of identifiable activating stress and possibly in relation to a larger distribution of adrenergic receptors in the basal segments of LV in this population [2, 12, 13]. The subjective experience of stress by woman in the course of delivery may be an additional factor contributing to occurrence of reverse SIC during caesarean section. SIC in the perioperative setting has been reported yet as well as vasoconstrictive substances such as ephedrine were suggested as triggering factors [14]. In our case, treatment of hypotension associated with spinal anaesthesia with ephedrine also appears to have precipitated SIC. Therefore adrenergic stimulants should be used with caution in treating maternal hypotension after spinal anaesthesia. However it is worth mentioning that hypotension is described as an undesirable result of central blockade and its prevalence is estimated as 30–90 % of all spinal anaesthesia. Standard methods of hypotension treatment in pregnancy are infusion of fluids and intravenous ephedrine administration, the use of corticosteroids is not recommended (their effect of action is too slow and weak). Ephedrine is easily adjustable and has a short duration of action so after all it appears to remain the first line option of treatment of hypotension associated with spinal anaesthesia. Moreover, taking into consideration that main aetiology of SIC appears to be the catecholamine- triggered myocyte injury, exogenous catecholamines in such patients with cardiogenic shock should be avoided. The use of inotropes and further activation of catecholamine receptors may worsen the clinical status and prognosis of these individuals. Unfortunately, due to persistent hypotension LV dysfunction, our patient received inotropic agents. This treatment could probably exacerbate or prolong the acute phase of the disease. According to current state of knowledge on Takotsubo syndrome of the Heart Failure Association of the European Society of Cardiology, in this case, treatment options should include mechanical support such as temporary LV assist devices, extracorporeal membrane oxygenation or if those option are not available low- dose of levosimendan infusion as a catecholamine sparing positive inotrope [15]. In the pathogenesis of SIC it is also suggested that lower estrogen levels may play a role [16]. In late pregnancy placenta produces high amounts of estradiol that decrease rapidly after placenta expulsion favouring a higher myocardium susceptibility to adrenergic stimuli. Consequently woman in the postpartum may represent another vulnerable group for SIC onset, especially inverted SIC.

Minatoguchi et al. identified a total of 18 (8 from Western countries) confirmed cases of symptomatic P- SIC [4]. Most women underwent caesarean delivery. Almost all electrocardiograms showed abnormalities and serum levels of cardiac enzymes were elevated. In 33 % cases, as well as in our, acute cardiopulmonary collapse occurred on the day of labour and symptoms were similar to those in ACS, PE or peripartum cardiomyopathy (PCM). In presented case computed tomography, as a diagnostic test of choice, excluded PE whereas coronary angiography has not confirmed ACS. PCM differs from typical SIC in terms of presentation and outcome and it does not present with a remarkable contraction pattern. Moreover recovery in PCM may be delayed of several months and is prevalent in less than 50 % of patients, whereas repeated TTE, among women with SIC, revealed normalized LV systolic function within 6 months in all reported cases [4, 17]. Furthermore in PCM myocardial late gadolinium enhancement can be present in cardiac magnetic resonance [18]. Therefore multimodality imaging may help to exclude or confirm the diagnosis of SIC. Echocardiography still remains preferred screening method to asses cardiac function. Bedside TTE seems to be a useful, non- invasive readily available diagnostic tool for the differential diagnosis of ACS, PE, PCM and SIC. Instantaneous recognition of SIC in the postpartum period has immediate therapeutic implications. Although SIC generally has a good prognosis, intensive heart failure therapy is required for successful convalescence.

## Conclusions

In conclusion, this case shows that inverted SIC may occur in young women, particularly in postpartum period, especially in combination with spinal anaesthesia and adrenergic stimulants administration.

On the basis of our experience we recommend the clinical awareness of possible diagnosis and further management of this unexpected variant of acute heart failure after caesarean delivery. Although initially the inverted stress- induced cardiomyopathy may seem dramatic, proper and accurate diagnosis usually leads to patients positive and successful outcome.

### Ethics, consent and permissions

I confirm that the study has been approved by Medical University of Lodz Ethics Committee.

### Consent to publish

I confirm that formal written consent to publish all participant’s data was obtained from individuals involved in the study.

## Abbreviations

ACS: acute coronary syndrome
LV: left ventricular
P-SIC: pregnancy associated stress- induced cardiomyopathy
PE: pulmonary thromboembolism
PMC: peripartum cardiomyopathy
SIC: stress- induced cardiomyopathy
TTE: transthoracic echocardiography
